# Belinostat exerts antitumor cytotoxicity through the ubiquitin‐proteasome pathway in lung squamous cell carcinoma

**DOI:** 10.1002/1878-0261.12064

**Published:** 2017-05-30

**Authors:** Li R. Kong, Tuan Z. Tan, Weijie R. Ong, Chonglei Bi, Hung Huynh, Soo C. Lee, Wee J. Chng, Pieter J. A. Eichhorn, Boon C. Goh

**Affiliations:** ^1^ Cancer Science Institute of Singapore National University of Singapore Singapore; ^2^ National Cancer Centre Singapore; ^3^ National University Cancer Institute Singapore; ^4^ Department of Hematology‐Oncology National University Hospital Singapore; ^5^ Department of Pharmacology Yong Loo Lin School of Medicine National University of Singapore Singapore

**Keywords:** histone deacetylase inhibitors, lung squamous cell carcinoma, MAPK inhibition, ubiquitin‐proteasome system

## Abstract

There have been advances in personalized therapy directed by molecular profiles in lung adenocarcinoma, but not in lung squamous cell carcinoma (SCC). The lack of actionable driver oncogenes in SCC has restricted the use of small‐molecule inhibitors. Here, we show that SCC cell lines displayed differential sensitivities to belinostat, a pan‐histone deacetylase inhibitor. Phosphoproteomic analysis of belinostat‐treated SCC cells revealed significant downregulation of the MAPK pathway, along with the induction of apoptosis. In cisplatin‐resistant cells that demonstrated aberrant MAPK activation, combined treatment with belinostat significantly inhibited cisplatin‐induced ERK phosphorylation and exhibited strong synergistic cytotoxicity. Furthermore, belinostat transcriptionally upregulated the F‐box proteins FBXO3 and FBXW10, which directly targeted son of sevenless (SOS), an upstream regulator of the MAPK pathway, for proteasome‐mediated degradation. Supporting this, suppression of SOS/ERK pathway by belinostat could be abrogated by inhibiting proteasomal activity either with bortezomib or with siRNA knockdown of *FBXO3/FBXW10*. Taken together, these preclinical data offer a novel understanding of the epigenetic mechanism by which belinostat exerts its cytotoxicity and supports the combination with cisplatin in clinical settings for chemorefractory SCC tumors.

**Abbreviations**
CI: combination index
FBXO3: F‐box protein 3
FBXW10: F‐box and WD repeat domain containing 10
HDAC: histone deacetylases
IC_50_: half‐maximum inhibitory concentrations
MAPK: mitogen‐activated protein kinases
NSCLC: non‐small‐cell lung cancer
PARP: poly(ADP‐ribose) polymerase
PI: propidium iodide
PXD101: belinostat
RT‐qPCR: quantitative real‐time polymerase chain reaction
SCC: squamous cell carcinoma
Scr: scrambled control siRNA
SOS: son of sevenless
STAT3: signal transducer and activator of transcription 3
UBE2C: ubiquitin‐conjugating enzyme E2 C

## 1 Introduction

Modification of histones through epigenetic dysregulation is an important oncogenic mechanism and has been exploited for cancer drug development. Histone deacetylases (HDAC) are reported to be overexpressed or aberrantly recruited by oncoproteins (Minucci and Pelicci, 2006), which subsequently leads to cancer initiation and progression (Glozak and Seto, 2007; Hess‐Stumpp, 2005). Studies involving the knockdown of HDAC1 have shown that loss of HDAC1 expression blocked mitosis in tumor cells (Senese *et al*., 2007), while HDAC3 silencing reduces the growth of colon cancer cells (Wilson *et al*., 2006). Furthermore, inhibition of HDACs sensitizes both chemotherapy and small‐molecule inhibitors in drug‐resistant cancer cells (Bangert *et al*., 2011; Chen *et al*., 2013; Sharma *et al*., 2010). HDAC inhibitors have been developed clinically, of which romidepsin, vorinostat, and belinostat have received regulatory approval for the treatment of cutaneous and peripheral T‐cell lymphoma (Mann *et al*., 2007; O'Connor *et al*., 2015; Piekarz *et al*., 2009). These studies emphasize the critical role of HDACs in tumor cells and implicate the potential of HDAC inhibitor as a potent antineoplastic agent.

However, single‐agent activity of HDAC inhibitors has been disappointing in solid tumors, likely contributed by significant clinical toxicities that preclude continuous exposure and dose escalation to adequate concentrations required for activity (Lane and Chabner, 2009). Moreover, unlike tyrosine kinase‐targeting agents, there is no available biomarker to select patients who are likely to respond to HDAC inhibitors treatment. Despite this, HDAC inhibitors still pose to be an interesting therapeutic regime when used in combination, especially in lung cancer. Vorinostat has been shown to improve clinical response when added to both squamous and nonsquamous non‐small‐cell lung cancer (NSCLC) patients treated with paclitaxel and carboplatin, despite not significantly improving the progression‐free and overall survival rates (Ramalingam *et al*., 2010). Moreover, metastatic lung cells could be resensitized to EGFR inhibitors with HDAC inhibitors (Witta *et al*., 2006). However, the role of HDAC inhibitors in tumors lacking actionable driver oncogenes, such as lung squamous cell carcinoma (SCC), has not yet been explored.

Belinostat (PXD101) is a hydroxamic acid‐type pan‐HDAC inhibitor that has demonstrated strong antineoplastic activity *in vitro* (Lin *et al*., 2013; Plumb *et al*., 2003; Qian *et al*., 2006). Importantly, when compared to other HDAC inhibitors, belinostat seemingly produces tolerable adverse effects, while hematological toxicity is rare (Gimsing *et al*., 2008; Ramalingam *et al*., 2009; Steele *et al*., 2008; Yeo *et al*., 2012). Indeed, belinostat has progressed to late‐stage clinical development in multiple hematologic and solid malignancies (Gimsing *et al*., 2008; Mackay *et al*., 2010; Ramalingam *et al*., 2009). Despite the mixed outcome obtained from these clinical evaluations, the well‐tolerable adverse effects of belinostat have encouraged the study of combination regimens.

The current study highlights the therapeutic efficacy of belinostat in lung SCC cells and characterizes the downstream molecular signaling in belinostat‐treated cells. We have previously identified activation of MAPK/ERK as a mechanism of resistance to cisplatin (Kong *et al*., 2015). The increase in ERK phosphorylation upon cisplatin treatment was shown to be regulated by son of sevenless (SOS) upstream. The current study demonstrates the promising chemosensitizing characteristic of belinostat in lung SCC through the suppression of MAPK activity. We further studied the mechanistic actions of HDAC inhibition by analyzing the global gene expression profiling, and identified the possible role of ubiquitin‐proteasome pathway in the regulation of SOS/MAPK signaling.

## 2 Materials and methods

### 2.1 Cell lines and reagents

All lung cell lines were obtained directly from the American Type Culture Collection (ATCC, Manassas, VA, USA). Lung SCC lines H226, H2170, H520, H596, and ChaGo‐k‐1 were maintained in RMPI‐1640 (Nacalai Tesque), Calu‐1 in McCoy's 5a (Nacalai Tesque, Kyoto, Japan), SK‐MES‐1 in DMEM (Nacalai Tesque), SW900 in Leibovitz’s medium (Gibco, Life Technologies, Carlsbad, CA, USA), H1869 in ACL‐4 (Gibco, Life Technologies), and H2066 in HITES medium (Gibco, Life Technologies). Normal lung fibroblast cells (MRC‐5, IMR‐90, and WI‐38) were cultured in EMEM (Gibco, Life Technologies). All media were supplemented with 10% fetal bovine serum, 2 mm l‐glutamine, 100 μg·mL^−1^ streptomycin, and 100 U·mL^−1^ penicillin. ACL‐4 and HITES were further supplemented with additional nutrients as recommended by the ATCC. All cell lines were authenticated with GenePrint^®^ 10 System (Promega, Fitchburg, WI, USA).

For inhibitor studies, belinostat (PXD101), bortezomib (Velcade), GDC0879, PD0325901, RDEA119, and GSK1120212 were obtained from Selleck Chemicals (Houston, TX, USA); cisplatin from Hospira (Lake Forest, IL, USA); MG‐132 from Sigma (St. Louis, MO, USA); and cetuximab was from Merck (Darmstadt, Germany).

### 2.2 Cell viability assay and synergism analysis

Cells were cultured (2000–4000 cells/well) on microtiter culture plates and treated with various concentrations of compounds for 72 h. At assay endpoint, cells were incubated with CellTiter 96^®^ AQ_ueous_ One Solution (MTS) solution (Promega) at 37 °C for 3 h. Absorbance was measured at 490 nm. All data points were set up with three replicates for each experiment. Percent cell proliferation was calculated relative to DMSO control‐treated cells.

The sigmoidal dose–response curve fitting for belinostat and the half‐maximum inhibitory concentration (IC_50_) were calculated by graphpad prism software (GraphPad Software, La Jolla, CA, USA). Combination index (CI) for belinostat and cisplatin was calculated based on Loewe additivity equation (Chou and Talalay, 1984), using the drug concentrations that resulted in the inhibition of cell viability between 50% and 80%, and tabulated at ED_50‐80_.

### 2.3 Proteome profiler antibody array

Whole‐cell lysates from vehicle‐ or belinostat‐treated cells were prepared 48 h after drug incubation and normalized according to protein concentrations. Cell lysates (250 μg) were mixed with biotinylated detection antibodies, and Proteome Profiler Phospho‐MAPK arrays (R&D Systems, Minneapolis, MN, USA) were performed. Chemiluminescence signal detection was performed, and densitometric data were analyzed with imagej (NIH, Bethesda, MD, USA). The analyses were performed by subtracting the background density signal, and positive control signals were used as internal control. Comparisons were made between the vehicle‐ and belinostat‐treated cells and expressed in fold change to vehicle control group.

### 2.4 Total protein extraction and western blotting

Vehicle‐ or compound‐treated cells were lysed 48 h after drug incubation with Cell Lytic buffer (Sigma; 150 mm NaCl, 0.41% bicine, 2% EDTA) supplemented with complete protease inhibitor cocktail (Roche, Basel, Switzerland) and phosphatase inhibitor cocktail (Roche). Protein concentration was quantified with BCA assay, and normalized samples were resolved with Bio‐Rad SDS/PAGE system on 8–12% protein gels. Signal detection was performed with chemiluminescence detection system (GE Healthcare, Chicago, IL, USA).

Poly(ADP‐ribose) polymerase (PARP), caspase 3, p‐ERK1/2 (Thr202/Tyr204), total ERK1/2, p‐p38 (Thr180/Tyr182), total p38, p‐BRAF (Ser445), p‐MEK1/2 (Ser217/221), total MEK1/2, SOS1, acetyl‐H3 (Lys9/14), p‐STAT3 (Ser727), total STAT3, UBE2C, HDAC1, HDAC2, HDAC3, HDAC4, HDAC5, Sirt1, β‐actin, and HRP‐conjugated anti‐rabbit were obtained from Cell Signaling (Danvers, MA, USA); SOS2 was purchased from Abcam (Cambridge, UK); FBXO3 was purchased from Sigma Aldrich (St. Louis, MO, USA); FBXW10 was purchased from Novus Biologicals (Littleton, CO, USA).

### 2.5 Apoptosis assay

Annexin‐V and propidium iodide (PI) staining was carried out according to the manufacturer's protocol with slight modifications (Thermo Fisher, Waltham, MA, USA). Briefly, 1–1.5 × 10^5^ cells were treated with belinostat for 48 h. On day of assay, both floating and adherent cells were collected. Annexin‐V‐APC (Thermo Fisher) was used as a probe for apoptosis, while PI (BD Biosciences, Franklin Lakes, NJ, USA) as an indicator for dead cells. Cells were stained in 100 μL Annexin‐V binding buffer containing both Annexin‐V‐APC and PI in the dark for 15 min at room temperature. The staining profile was collected and analyzed using LSR II Flow Cytometer (BD Biosciences).

### 2.6 Data preprocessing of Affymetrix microarray gene expression

Microarray gene expression data of eight pairs of control belinostat‐treated lung cancer cell lines were performed on Affymetrix Gene 1.0 ST and are deposited in Gene Expression Omnibus with the accession ID GSE85979. Robust multichip average normalization was performed using Affymetrix Powertool v1.18.0 (Santa Clara, CA, USA). All arrays were checked for quality: dabg, PM_MEAN, POS_vs_NEG_AUC, and passed the recommended quality criteria.

### 2.7 RT^2^ profiler and quantitative real‐time polymerase chain reaction (RT‐qPCR)

Total RNA was prepared using RNeasy according to the manufacturer's protocol (Qiagen, Venlo, Netherlands). Reverse transcription PCR for the conversion of cDNA was performed according to the recommended protocols for *Taqman* ^*®*^ (Applied Biosystems, Foster City, CA, USA) and RT^2^ First Strand kit (SABiosciences, Venlo, Netherlands), respectively.

The human Ubiquitination Pathway RT^2^ Profiler PCR array (SABiosciences) was used to assess the regulation of ubiquitin‐proteasome‐related genes upon belinostat treatment. The expressions of 84 key genes associated with the ubiquitination pathway were quantified according to the manufacturer's protocol. Data shown represent the mean of two replicates and were normalized to multiple housekeeping genes. qPCR was performed using either SYBR Green or *Taqman* ^*®*^ system, and the primer sequences are listed in Table S1. GAPDH was applied as housekeeping gene.

### 2.8 Anchorage‐independent soft agar assay

Soft agar was mixed with culture media to form multiple agar layers: a bottom layer with 0.6% agar; a middle layer with 0.36% agar and resuspended with 5000–10 000 cells; and a top layer with complete media containing belinostat, cisplatin, or belinostat / cisplatin combination at various doses. Colonies were allowed to form for 2–4 weeks. On assay endpoint, viable colonies were stained with MTT solutions (5 mg·mL^−1^ in PBS) at 37 °C for 4 h. Images of each well were acquired with Epson V330 Photo scanner. The number and size of the colonies were analyzed and quantified using imagej (NIH). Percentage cell colony formation was calculated relative to DMSO control‐treated cells.

### 2.9 RNA interference

For gene knockdown, Stat3 was obtained from Ambion (Thermo Fisher Scientific, Waltham, MA, USA). FBXO3 siRNA (sequence: 5′‐GACGAUUAUCGAUGUUCAUTT‐3′), FBXW10 siRNA (sequence: 5′‐CUCCGGUCUAUAUCCGAAATT‐3′), and AllStar scrambled control siRNA (scr siRNA) were obtained from Qiagen. Transfection (50 nm siRNA for each target in each reaction) was conducted with JetPRIME reagent (Polyplus Transfection, Strasbourg, France).

### 2.10 Xenograft studies

All *in vivo* studies adhered to the Institutional Animal Care and Use Committee (IACUC) guidelines on animal use and handling. Calu‐1 xenograft model was established and maintained in 8‐ to 10‐week‐old female SCID mice. In brief, 10 × 10^6^ Calu‐1 cells in 100 μL of PBS were injected subcutaneously into the flanks of each mice. Treatment began when the tumor sizes reached approximately 200 mm^3^; the mice were assigned into four stratified groups based on average tumor volume: vehicle (1% w/v polysorbate 80), cisplatin, belinostat, belinostat + cisplatin (*n *= 5 animals/10 tumors per group). Cisplatin was provided in 0.9% NaCl and given at 4 mg·kg^−1^ by intraperitoneal (ip), once weekly for 3 weeks. Belinostat was formulated in 30% (w/v) Captisol and 30% (w/v) PEG300 at 40 mg·kg^−1^ and given via PO (orally), 5 days a week, until mice were sacrificed. Tumor growth was monitored and measurements were taken three times weekly (tumor volume was estimated by [length × width × width × 3.14159/6]). Bodyweight at sacrifice and tumor samples were collected upon reaching experiment endpoint when mice were sacrificed 3 weeks after treatment commenced.

### 2.11 Histone extraction

Vehicle‐ or belinostat‐treated cells were lysed 48 h after drug incubation with Triton extraction buffer (PBS containing 1% Triton X‐100 (v/v), 10 mm Tris/HCl, 50 mm sodium bisulfite, 100 mm MgCl, and 8.6% sucrose pH 6.3) and subjected to Dounce homogenization. Cell nuclei were collected by centrifugation, and pellets were washed several times with Triton extraction buffer. The histones were acid‐extracted for an hour at 4 °C with 0.4 N sulfuric acid. Next, the supernatants were collected with centrifugation and mixed with acetone overnight at −20 °C. Finally, histones were pelleted at 14 000 rpm, air‐dried at room temperature, and dissolved in water.

### 2.12 Statistical analysis

All experiments were conducted three times unless stated otherwise. Results are presented as mean ± SD. Statistical analysis for the comparison between two groups was conducted using Student's *t*‐test, while comparisons between multiple groups were made using ANOVA. All tests were two‐sided, and the significance level was set at *P *< 0.05.

## 3 Results

### 3.1 Characterizing the antitumor effects of belinostat in lung SCC cells

**Figure 1:**
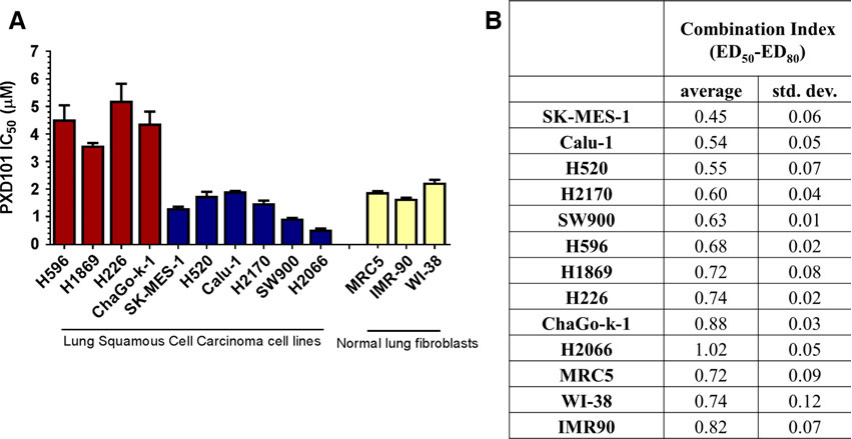
Belinostat demonstrates efficacy in lung squamous cell carcinoma (SCC) cells. (A) Lung SCC cell lines and normal lung fibroblast cell lines were treated with belinostat (PXD101) for 72 h, and cell viability was determined with CellTiter assay. Data are represented as mean IC_50_ ± SD (*n *= 3). (B) Lung SCC cell lines and normal lung fibroblast cell lines were treated with belinostat (PXD101) and cisplatin for 72 h using constant ratio at IC_50 (_ _PXD__101)_/IC_50 (_ _CDDP__)_ combination, and cell viability was determined with CellTiter assay. The combination index (CI) values of belinostat and cisplatin between effective doses of 50–80% were calculated from isobologram analysis. Values were shown as average CI ± SD (*n *= 3). Combination index of CI < 0.8 indicates synergism, 0.8 < CI < 1.2 indicates additive effect, and CI > 1.2 indicates antagonism.

To determine the sensitivity of HDAC inhibitors in SCC cells, we screened a panel of 10 lung SCC cells and three normal fibroblast cells against belinostat to determine the half‐maximum inhibitory concentrations (IC_50_). All three lung fibroblast cells were shown to have similar toxicity to belinostat (IC_50 _~ 3 μm). In order to avoid normal tissue toxicity, we defined lung SCC cells with IC_50_ < 3 μm as belinostat sensitive, while those above this cutoff as belinostat resistant (Fig. 1A). Interestingly, lung SCC cells (H2066, SW900, H2170, Calu‐1, H520, and SK‐MES‐1), which we previously demonstrated to exhibit decreased sensitivity to cisplatin, were highly sensitive to belinostat (Kong *et al*., 2015). The reciprocally inverse sensitivity among belinostat and cisplatin suggested the potential for synergistic combination. Our combination index (CI) analysis revealed that belinostat synergized with cisplatin in most cell lines, with the most pronounced synergy observed in SK‐MES‐1, Calu‐1, and H520 cells (CI < 0.6) (Fig. 1B). However under anchorage‐independent growth, belinostat showed additive effect when used in combination with cisplatin (Fig. S1A), with average CI (ED_50_‐ED_80_) in between 0.82 and 1.19 (Fig. S1B). Taken together, these results suggest that belinostat regulates chemosensitivity in lung SCC cells, and are consistent with previous reports on HDAC inhibitors (Ramalingam *et al*., 2010).

### 3.2 Suppression of MAPK signaling is associated with sensitivity to belinostat

HDAC inhibitors are epigenetic modulators that often induce growth inhibition in cancer cells. To investigate the antitumor mechanisms in the context of lung SCC, we examined the transcriptomic and kinomic changes in belinostat‐treated cells. Gene expression profiling was conducted to compare the significantly altered genes after exposure to belinostat. However, transcriptomic analyses offered little insight into the mechanism of action of belinostat, as gene ontology and pathway enrichment analyses could not identify differentially regulated signaling pathway across the belinostat‐sensitive (H2066, SW900, H2170, and Calu‐1) and belinostat‐resistant (H226, H596, ChaGo‐k‐1, and H1869) cells (data not shown).

**Figure 2:**
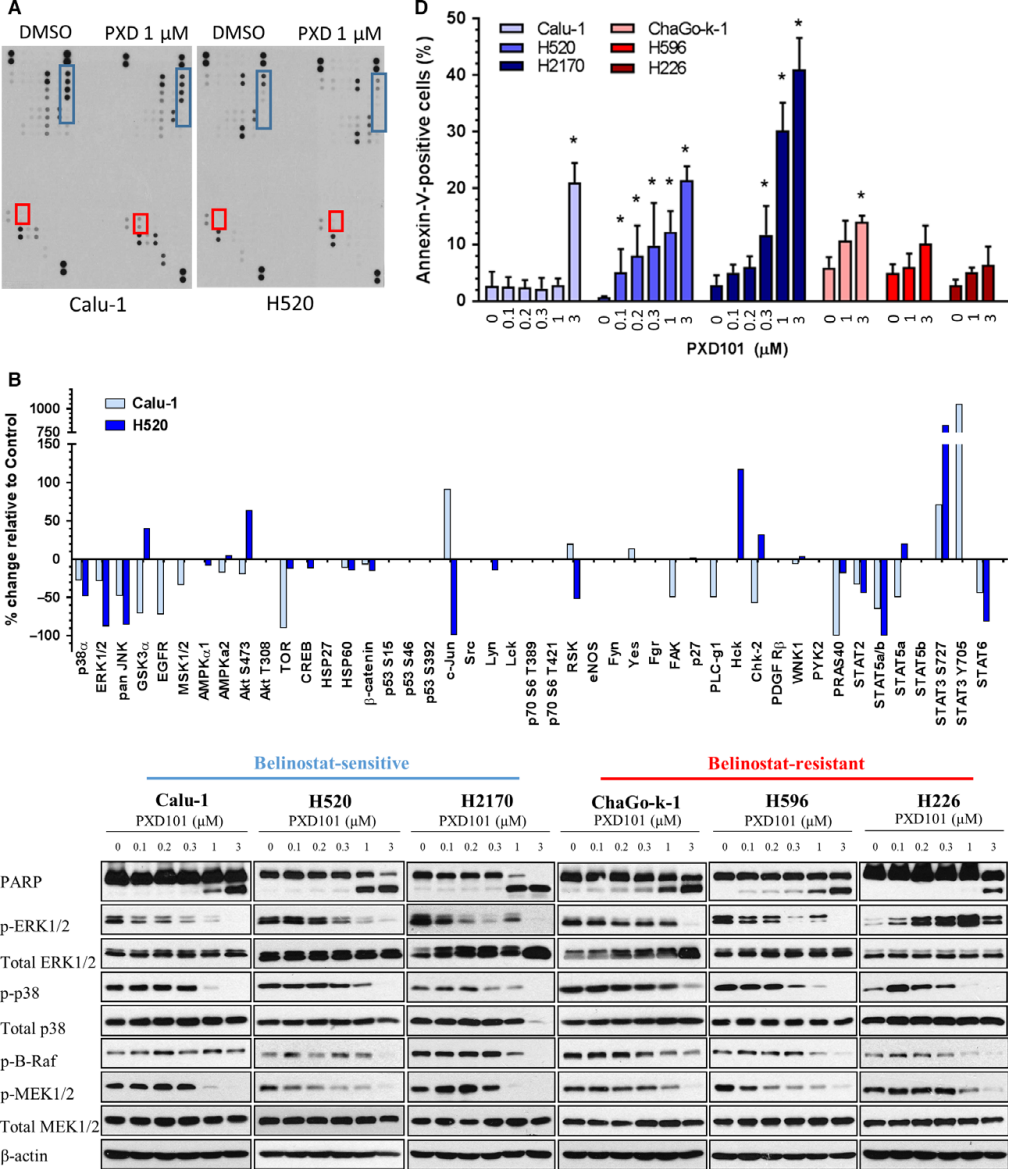
Belinostat suppresses MAPK signaling and triggers apoptosis in lung squamous cell carcinoma (SCC). (A) Calu‐1 and H520 cells were treated with vehicle or 1 μm belinostat (PXD101) for 48 h with the protein lysates harvested for phosphokinase profiling. Dots representing consistently elevated targets are highlighted in red (STAT3), and suppressed kinases are highlighted in blue (p38, ERK1/2, JNK). (B) Densitometry analysis on the phosphokinase profiling was performed as described in Materials and methods. The average values of the significantly regulated duplicate were shown. (C) Cell lysates were harvested from lung SCC cells after treatment with increasing doses of belinostat (0.1, 0.2, 0.3, 1, 3 μm). Immunoblotting was performed to evaluate the changes in phosphorylated protein levels of the targets identified in 1D (ERK1/2, p38, B‐Raf, MEK1/2) as well as PARP. β‐Actin shown as loading control. (D) Lung SCC cells were treated with vehicle or belinostat (0.1, 0.2, 0.3, 1, 3 μm for Calu‐1, H520, and H2170; 1, 3 μm for ChaGo‐k‐1, H596, and H226) and stained with Annexin‐V/propidium iodide (PI). The percentage of Annexin‐V‐positive cells was shown as mean ± SD. *P < 0.05.

Next, phosphokinases arrays covering multiple crucial phosphoproteins were performed on belinostat‐sensitive H520 and Calu‐1 cells (Fig. 2A). The signal intensities of each pair of duplicate targets were quantified, and the expression levels in belinostat‐treated cells were compared to the DMSO control. Across these two cell lines, perturbations were observed in the phosphoprotein expressions of p38, ERK1/2, JNK, GSK3α, AMPK, AKT, TOR, c‐JUN, RSK, CHK‐2, PRAS40, STAT2, STAT3, STAT5, and STAT6 (Fig. 2B). Interestingly, belinostat consistently suppressed the phosphorylation of MAP kinases (p38, ERK, and JNK), while it increased STAT3 phosphorylation, in both Calu‐1 and H520 cells (Fig. 2B).

We sought to verify the changes in kinase phosphorylation by western blotting in independent cell lysates of belinostat‐sensitive (Calu‐1, H520, H2170) and belinostat‐resistant cell lines (H226, H596, ChaGo‐k‐1). SCC cells were treated with increasing doses of belinostat that fall within the clinically achievable plasma concentration (Yeo *et al*., 2012). Of note, belinostat treatment abrogated the phosphorylation of ERK1/2 and p38 in all SCC cells, except for the highly resistant H226 cells (Fig. 2C). Consistently, upstream kinases of ERK1/2, MEK1/2, and B‐Raf were inhibited by increasing doses of belinostat. As activation of the MAP kinase pathway limits the induction of apoptosis, we looked to see whether apoptosis was induced in belinostat‐treated cells. As expected, treatment with belinostat increased PARP cleavage, a marker of apoptosis (Fig. 2C, top). Furthermore, belinostat dose‐dependent treatment significantly increased Annexin‐V‐positive populations in belinostat‐sensitive cells (Calu‐1, H520, and H2170), but not in belinostat‐resistant cells (ChaGo‐k‐1, H596, and H226) (Fig. 2D).

The proapoptotic Bim protein has been well characterized to be negatively regulated by MAPK signaling (Luciano *et al*., 2003). We next asked whether Bim is involved in belinostat‐induced apoptosis, and showed that belinostat significantly elevated Bim expression in belinostat‐sensitive cells (Calu‐1, H520), but to a lesser extent in H226 cells (Fig. S4A). Accordingly, silencing of Bim partially reduced apoptosis in belinostat‐treated Calu‐1 and H520 cells, as shown by the decrease in Annexin‐V‐positive cells (Fig. S4B). Collectively, these data suggest that treatment with belinostat induces cell death in lung SCC cells, possibly through the suppression of MAPK signaling and Bim regulation.

Interestingly, STAT3 phosphorylation was enhanced in belinostat‐treated Calu‐1 cells (Fig. S2A), likely through the increase in transcripts of STAT3‐related cytokines (Fig. S2B). This is in concordance with data from a recent study showing that MEK inhibition triggers autocrine activation of JAK/STAT3 pathway in oncogene‐addicted cells as a positive feedback mechanism (Lee *et al*., 2014). Likewise, treatment with the MEK inhibitors (PD0325901, RDEA119, and trametinib), but not the B‐Raf inhibitor (GDC0879) or the EGFR inhibitor (cetuximab), significantly suppressed ERK activation and enhanced STAT3 phosphorylation in Calu‐1 cells (Fig. S2C). However, in the context of SCC cells, STAT3 activation likely does not provide a significant survival advantage as STAT3 silencing did not augment belinostat‐induced PARP cleavage in Calu‐1 cells (Fig. S2D). Nevertheless, these observations strengthen our claim that belinostat treatment attenuates MAPK signaling, which in return triggers positive STAT3 activation as a feedback mechanism.

### 3.3 Belinostat blocks SOS/MAPK activation in cisplatin‐resistant cells

**Figure 3:**
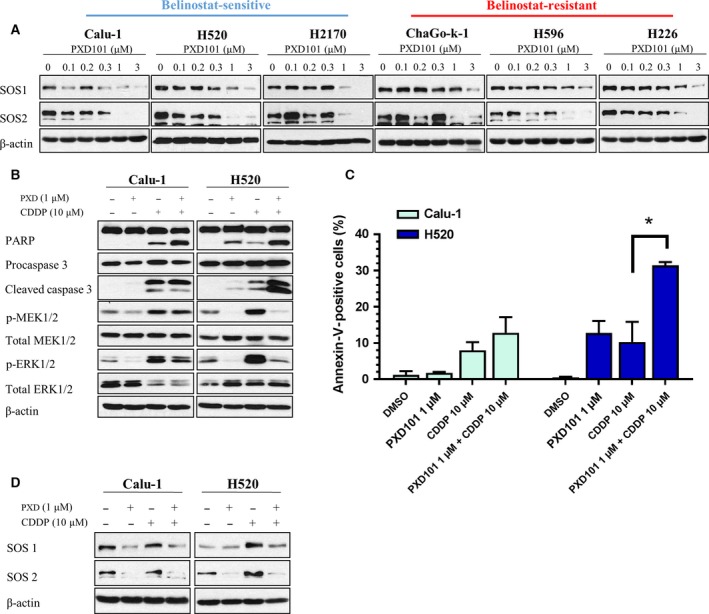
Suppression of MAPK signaling by belinostat is associated with downregulation of SOS and leads to synergistic cytotoxicity in cisplatin‐resistant cells. (A) Cell lysates were harvested from lung SCC cells after treatment with increasing doses of belinostat (PXD101) (0.1, 0.2, 0.3, 1, 3 μm) to evaluate the changes in SOS1 and SOS2. The combinatory effects of belinostat (1 μm) and cisplatin (10 μm) on (B) PARP, caspase 3, MEK, and ERK; and (D) SOS1 and SOS2 were examined with immunoblotting, (C) while the effects on apoptosis were evaluated with Annexin‐V/propidium iodide (PI). β‐Actin shown as loading control. Data were shown as mean ± SD. *P < 0.05.

Son of sevenless is a guanine nucleotide exchange factor that regulates the activation of Ras‐GTPases and subsequently induces MAPK signaling (Gureasko *et al*., 2008; Rogge *et al*., 1991). It has been shown previously that SOS upregulation leads to cisplatin resistance in lung SCC cells through MAPK/ERK pathway activation (Kong *et al*., 2015). As belinostat demonstrated strong inhibition of MAPK signaling, we therefore investigated the expression status of SOS upon exposure to belinostat. We observed suppression of SOS proteins, particularly SOS2, in all SCC cell lines irrespective of their belinostat sensitivity (Fig. 3A). We therefore postulated that tumor cells with aberrant activation of MAPK signaling, such as those resistant to cisplatin, will be sensitized by belinostat. Cisplatin was used at a concentration (10 μm) below the maximum tolerated dose (van Hennik *et al*., 1987). Accordingly, we showed that combined treatment with 1 μm belinostat significantly suppressed cisplatin‐induced ERK1/2 phosphorylation in SCC, particularly H520 cell line (Fig. 3B). Furthermore, combined treatment with cisplatin and belinostat resulted in increased apoptosis as demonstrated by the augmentation in PARP and caspase 3 cleavage (Fig. 3B), together with potentiation in Annexin‐V‐positive cells (Fig. 3C). It is of note that with 1 μm belinostat, synergistic cytotoxicity was more significant in H520 cells. This potentiation in cytotoxicity positively correlated with the extent of p‐ERK1/2 suppression and was associated with downregulation of both SOS1 and SOS2 upon combined treatment with cisplatin and belinostat (Fig. 3D). Collectively, these data suggest a potential chemosensitization effect of belinostat through the inhibition of cisplatin‐induced SOS/MAPK activation in lung SCC cells.

**Figure 4:**
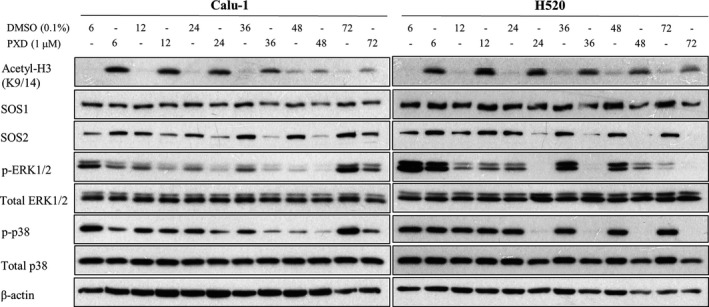
Belinostat induces acetylation of histone H3 and downregulates MAPK pathway progressively. Kinetic studies of belinostat (PXD101) treatment (1 μm) were performed over 72 h (6, 12, 24, 36, 48, 72 h) in Calu‐1 and H520 cells. The effects on acetylated histone H3, SOS1, SOS2, ERK, and p38 were evaluated with immunoblotting. β‐Actin shown as loading control.

To further elucidate the molecular underpinnings of SOS downregulation through HDAC inhibition by belinostat, we hypothesize that histone acetylation by belinostat regulates transcriptional events leading to SOS downregulation. Belinostat significantly induced acetylation of histone H3, as early as 6 h post‐treatment (Fig. 4, top). This observation was accompanied by the downregulation of SOS and decreased phosphorylation of MAP kinases within 48 h after belinostat treatment in both Calu‐1 and H520 cells (Fig. 4, bottom). In concordance with the reduction in acetylated H3, the inhibitory effects on MAPK signaling faded at 72 h post‐treatment in Calu‐1 cells. These temporal changes indicate the possibility of epigenetic‐regulated post‐transcriptional events that could impact the dynamics of SOS/MAPK signaling.

### 3.4 Induction of the ubiquitin ligases *FBXO3* and *FBXW10* in response to belinostat treatment in lung SCC

**Figure 5:**
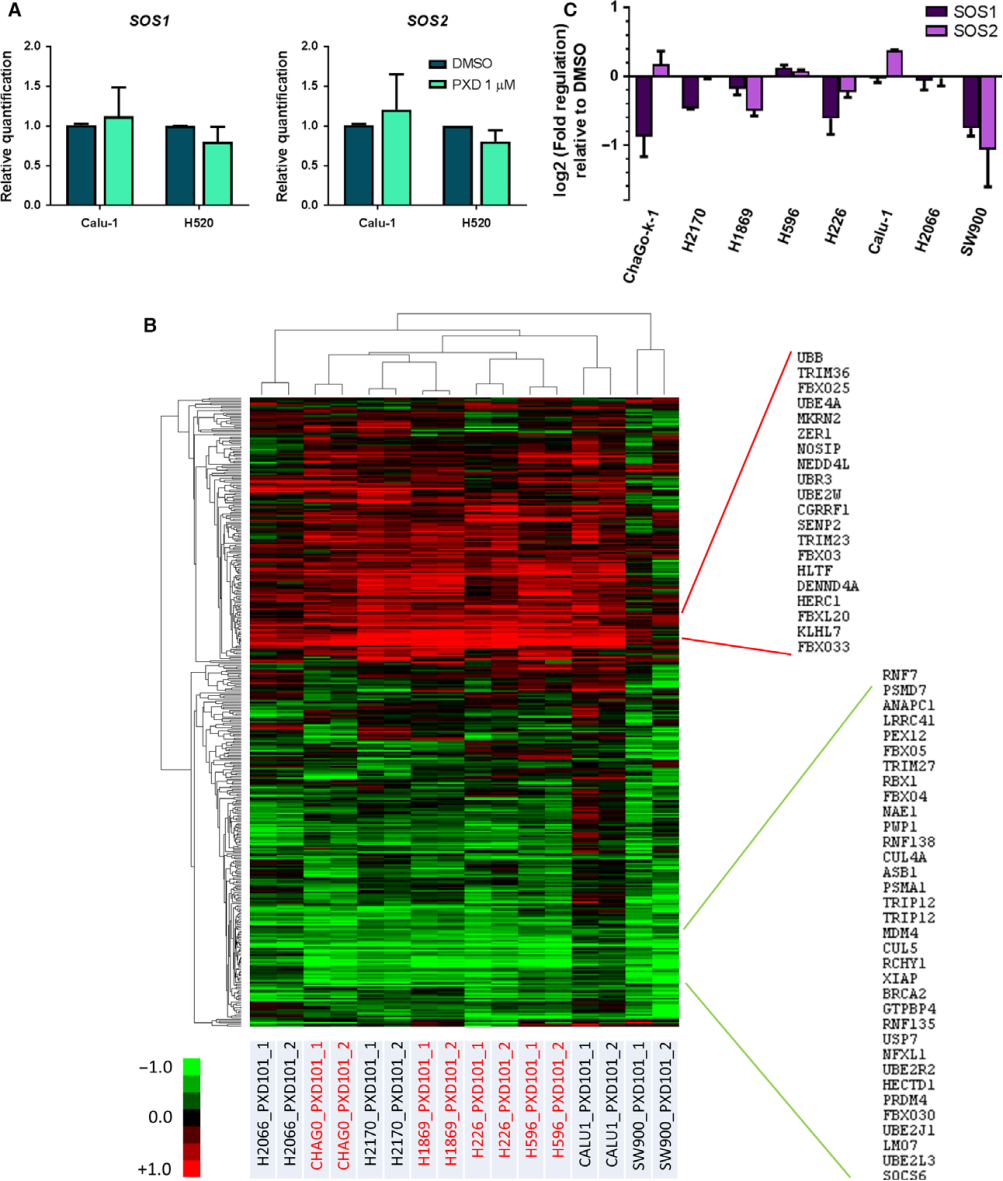
Ubiquitin‐related genes, but not *SOS 1* and *2*, are transcriptionally regulated by belinostat in lung squamous cell carcinoma (SCC) cells. (A) Real‐time quantitative PCR (qPCR) was conducted to quantify the mRNA expression of *SOS1* and *SOS2* after vehicle or belinostat (PXD101) (1 μm) treatment. Data were represented as average relative quantification ± SD (*n *= 3). (B) Heat map represents the gene expression of altered genes (fold change > 1.5) identified by Affymetrix microarrays across all lung SCC cells (H596, H1869, H226, and ChaGo‐k‐1, Calu‐1, H2170, SW900, and H2066) treated with belinostat at respective IC_50_. Consistently upregulated genes were highlighted in red, and downregulated genes in green. (C) Expressions of both *SOS1* and *SOS2* from the same array were tabulated in the bar chart as average fold regulation ± SD.

We first investigated the possibility of transcriptional perturbations through histone acetylation induced by belinostat to explain SOS downregulation. However, *SOS1* and *SOS2* mRNA expressions were not reduced following belinostat treatment (Fig. 5A). An alternative mechanism of SOS downregulation involving proteasomal degradation was explored. Through global gene expression analysis of belinostat‐treated cells, we derived gene sets to determine the possible involvement of ubiquitin‐proteasome pathway in the suppression of SOS in belinostat‐treated cells. Gene sets comprising ubiquitin‐related genes (657) as annotated by Molecular Signature Database (Msigdb.v5.0) were compiled and mapped to transcriptional changes in SCC cell lines induced by exposure to belinostat for 8 h. Expression values were derived relative to the DMSO control samples. The transcriptomic profiles of both belinostat‐tolerant (H226, H596, ChaGo‐k‐1, H1869) and belinostat‐sensitive (Calu‐1, H2170, SW900, H2066) cells were mapped to the curated and computational gene sets (GSEA c2, c4, and c5) (Table S2). Among the genes that appeared in the Affymetrix Gene 1.0ST platform, we filtered out and retained those that were regulated by a log2 fold change of > 0.5 in at least one cell line, and performed supervised cluster analysis (Fig. 5B). Interestingly, an ubiquitin‐related gene signature was significantly affected upon HDAC inhibition in both sensitive and resistant cell lines with 391 altered genes, but no apparent pattern that could be correlated with drug response. Of these, we identified clusters of genes that were consistently upregulated (54) or downregulated (91) upon belinostat treatment across all SCC cells. There was no significant perturbation of *SOS1* and *SOS2* in SCC cells within the same transcriptomic dataset, except for SW900 (Fig. 5C), which is consistent with the qPCR analysis shown in Fig. 5A.

**Figure 6:**
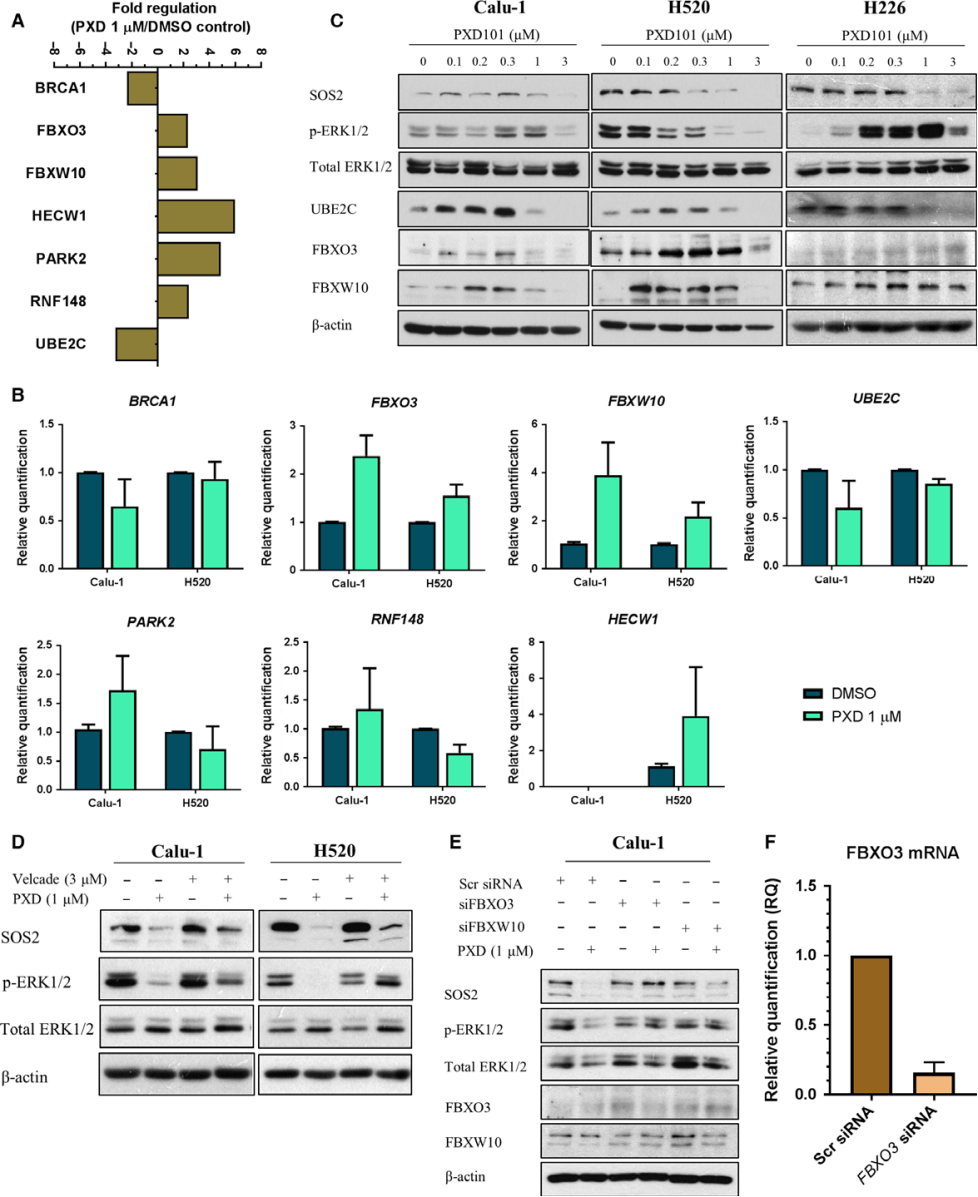
Regulation of ubiquitin gene expression profile upon belinostat treatment is associated with suppression of SOS/MAPK and could be rescued by bortezomib treatment. (A) Calu‐1 cells were treated with vehicle or belinostat (PXD101) (1 μm). Total mRNA transcript levels of 84 key ubiquitination genes were assessed with RT^2^ Profiler PCR Array. Data were presented as bar chart representing the relative value of the significantly altered genes (> twofolds) as compared to vehicle (*n *= 1). (B) The significantly altered genes identified from RT^2^ Profiler were verified with real‐time quantitative PCR (qPCR) in Calu‐1 and H520 cells treated with vehicle or belinostat (PXD101) (1 μm). Data were represented as average relative quantification ± SD (*n *= 3). (C) The genes regulated in the similar manner identified from qPCR were verified with immunoblotting in Calu‐1, H520, and H226 cells after treatment with vehicle or belinostat (1 μm). (D) Immunoblotting was performed to evaluate the effects of bortezomib (3 μm) on SOS2 and p‐ERK1/2 in belinostat‐treated cells. (E) Immunoblots show the effects of scrambled (Scr), FBXO3, or FBXW10 siRNA knockdown on affecting the modulation of belinostat on SOS/MAPK in Calu‐1 cells. (F) Knockdown efficiency of *FBXO3* in Calu‐1 cells was confirmed with RT‐qPCR (*n* = 2). Fifty nanomolar siRNA was used per transfection. β‐Actin shown as loading control.

We verified the clusters of gene panels derived from the microarray using RT^2^ Profiler PCR Array specifically targeting the expression of well‐established ubiquitination‐related genes encoding for E1, E2, and E3 enzymes. The gene expression changes were measured in Calu‐1 cells 24 h after belinostat treatment, when the inhibition of SOS/MAPK signaling was first observed (Fig. 4). The significantly altered genes were identified from the array (Fig. 6A) and validated by qPCR in both Calu‐1 and H520 cells (Fig. 6B). Among these genes, *FBXO3*,* FBXW10*, and *UBE2C* were identified as commonly altered genes (Fig. 6B). Moreover, exposure to 1 μm belinostat increased protein expressions of FBXO3 and FBXW10, but decreased UBE2C, in both Calu‐1 and H520 cells, providing concordance to the RT^2^ gene expression profile (Fig. 6C). Similar observations were made in belinostat‐resistant H226 cells (Fig. 6C). Taken together, the observations suggest a significant mechanism of functional protein regulation that occurs through epigenetic modulation of ubiquitin‐proteasome pathways by belinostat treatment. Moreover, these results may explain the downregulation of MAPK signaling through ubiquitination and degradation of SOS proteins.

To further validate this hypothesis, expression of SOS1/2 and ERK phosphorylation was studied after belinostat treatment after pharmacological inhibition of proteasomal degradation by bortezomib. The kinetic studies for bortezomib have been previously reported, with strong dose‐dependent inhibition on 20S proteasome activity observed within 24 h after treatment (Adams *et al*., 1999). Here, we combined 3 μm bortezomib in belinostat‐treated cells and demonstrated a rescue in the SOS2 expression and prevented ERK1/2 dephosphorylation (Fig. 6D). Importantly, similar results were observed in Calu‐1 cells transfected with siRNA targeting either *FBXO3* or *FBXW10* (Fig. 6E,F). These results strengthen our claim that suppression of SOS/MAPK is associated with ubiquitin‐mediated proteasomal degradation pathway following belinostat treatment.

## 4 Discussion

The mechanism of synergistic activity of HDAC inhibitor with chemotherapy is poorly understood. Several mechanisms have been proposed, including upregulation of tumor suppressor genes like p53, as well as inhibition of the aggresome (Condorelli *et al*., 2008; Mishima *et al*., 2015). Aberrations in the transcriptome of tumor cells are often controlled by chromatin remodeling and histone modifications (Glozak and Seto, 2007; Hess‐Stumpp, 2005). HDACs are often inappropriately expressed in cancer cells, with contentious roles in the development of oncogenesis. For instance, class II HDACs have been shown to repress oncogenes, and the reduced expression of HDAC5 and HDAC10 favored malignant progression in NSCLC (Osada *et al*., 2004). On the contrary, high expression of HDAC1 has been linked with poor prognosis in patients with lung adenocarcinoma (Minamiya *et al*., 2011). However, which HDACs are coregulated to augment cytotoxicity to cancer cells, particularly here in lung SCC tumors, remains elusive. We propose that belinostat synergizes cisplatin in platinum‐resistant SCC cells through upregulation of genes that regulate ubiquitin‐mediated proteasomal degradation leading to disruptions of key survival signaling molecules.

We initially screened a panel of 10 SCC cells and revealed a range of sensitivities to belinostat. Increasing doses of belinostat induced apoptosis in SCC cells, which is consistent with previous observations analyzing the effects of other HDAC inhibitors (Moore *et al*., 2004; Zhang *et al*., 2004). It is important to note that these differential sensitivities were not concordant with basal expression of HDACs, apart from HDACs 3 and 6 (Fig. S3); hence, alternative mechanisms were postulated to be responsible for the efficacy of belinostat in respective SCC cells. Through a phosphoproteomic profiling screen, we found that belinostat has a strong inhibitory effect on MAPK signaling (ERK1/2 and p38). Mechanistic studies demonstrated that various MAP kinases (MEK and B‐Raf) exhibited decreased phosphorylation levels, and expression of SOS was reduced following belinostat exposure. In contrast, STAT3 activity was enhanced. Lee *et al*. (2014) have previously demonstrated that MEK inhibition induces activation of STAT3 through a feedback mechanism, therefore supporting a strong mechanistic link between HDAC inhibition and MAPK signaling pathways (Vultur *et al*., 2014).

The expanding understanding on the chromatin architecture has described the post‐translational modifications of histone tails as a targeted therapeutic approach. HDAC inhibitors are known to augment the acetylation of lysine residues associated with histones and promote a relaxed chromatin structure to perturb gene transcriptions as a mean of shifting equilibrium toward proapoptosis (Moore *et al*., 2004; Struhl, 1998). We hypothesize that this could indirectly affect expression of SOS. While HDAC inhibitors have been reported to resensitize erlotinib‐resistant cells by transcriptionally reducing *EGFR* expression (Chen *et al*., 2013; Liffers *et al*., 2016), SOS mRNA levels were found to be unchanged following the addition of belinostat even though belinostat dose dependently induced acetylation of histones H3 and H4 (Fig. S5). However, overall SOS1 and SOS2 protein expression was reduced in belinostat‐treated cells, suggesting that belinostat may regulate SOS protein expression through a post‐transcriptional event. The lack of consistency between the protein and mRNA expressions of SOS prompted us to investigate more closely the transcriptional perturbations elicited by belinostat in SCC cells. In doing so, we identified a potential role of the ubiquitin‐proteasome pathway in these effects.

Collectively, transcriptional profiling of microarray data with subsequent RT‐qPCR validation showed enhanced expressions of a number of ubiquitin‐proteasome genes including members of the F‐box protein family (*FBXW10*,* FBXO3*) and E3 ligase components (*FBXO3*,* BRCA1*), as well as reduced expression of the ubiquitin‐conjugating enzyme (*UBE2C*), after belinostat treatment. The role of F‐box proteins has been implicated as an important component in tumor progression and development due to their direct regulation on various oncogenic signaling, and pertinently, they have been demonstrated to be epigenetically regulated. For instance, *FBXW10* gene is found to be hypermethylated in clear cell renal cell carcinoma (Wang *et al*., 2015), while FBXO3 influences TGF‐β signaling by targeting SMURF1 for proteasomal degradation (Li *et al*., 2015). This epigenetic regulation of the ubiquitin‐proteasome pathway relevant to oncogenesis affords therapeutic opportunity by HDAC inhibition and possibly DNA methylation inhibitors, and our work in lung SCC cells demonstrates this. We further validated this using both chemical and genetic means whereby the depletion of SOS by belinostat was prevented through the addition of the proteasomal inhibitor bortezomib or siRNA knockdown of *FBXO3* and *FBXW10*. However, it is important to note that combination therapies using inhibitors of proteasomes and HDACs are currently being explored (Bhatt *et al*., 2013; Pei *et al*., 2004). Similarly, we demonstrate that, despite observing the partial restoration of SOS/MAPK at early time point, prolonged exposure to bortezomib/belinostat combination leads to cell death (data not shown), which is likely due to the inhibition of aggresome formation in cells treated with bortezomib (Mishima *et al*., 2015). Nonetheless, we showed that silencing of either FBXO3 or FBXW10 could partially rescue cell viability under exposure to 1 μm belinostat in H520, but not in H226 cells, thus highlighting the role of F‐box protein in facilitating the cytotoxicity of belinostat (Fig. S6). Further studies will be required to elucidate the crosstalk between HDAC inhibition and the proteasome system.

Platinum‐based chemotherapies remain as the standard‐of‐care treatment for the past decades in lung SCC (Wang and Lippard, 2005), while small‐molecule kinase inhibitors have demonstrated limited efficacy to date, thus emphasizing the urgent need for a more effective treatment strategy for this disease. The present study shows that a belinostat/cisplatin combination is broadly effective across a panel of lung SCC cell lines, with a strong inhibition of MAPK and SOS. Our group has previously described the significance of SOS/MAPK activation in cisplatin resistance (Kong *et al*., 2015). On the basis of this study, we speculate that belinostat treatment promotes ubiquitin‐proteasome activity in SCC cells; however, cisplatin‐resistant cells that exploit SOS/MAPK activation for survival are more susceptible to belinostat treatment. The strong synergistic cytotoxicity indicates the potential of belinostat as a chemosensitizing agent especially in the treatment for chemorefractory tumors. Nonetheless, clinical trials on HDAC inhibitors have yet to achieve superior progress in advanced solid malignancies (Bradley *et al*., 2009; Mackay *et al*., 2010), largely due to the poor bioavailability and potency (Elaut *et al*., 2007). Our preliminary xenograft studies have shown a lack of antitumor activity when a tolerable dose of belinostat (40 mg·kg^−1^) was used singly or in combination with cisplatin (Fig. S7A–D). This could be reasoned by the poor pharmacokinetics of belinostat, as shown by the failure to induce histone H3 acetylation in the tumor mass (Fig. S7E). Therefore, the clinical application of belinostat still requires much fine tuning, both in terms of drug delivery and in terms of therapeutic window.

## 5 Conclusion

In summary, we have described a novel mechanism of belinostat sensitivity against lung SCC, a disease in which small‐molecule inhibitors have mostly failed due to the lack of actionable driver oncogenes. This mechanism involves the disturbance of the expressions of ubiquitin‐related proteins, which influences the activity of key survival signals, leading to an increase in cellular apoptosis. In the context of SCC, belinostat treatment triggers the proteasomal degradation of SOS proteins and downregulates the downstream MAPK signaling. Interestingly, HDAC inhibitors have been previously implicated in the depletion of mutant p53 through the transcriptional induction of *MDM2*, an E3 ubiquitin ligase that negatively regulates p53, instead of directly affecting *TP53* transcription (Blagosklonny *et al*., 2005). Likewise, a similar finding was earlier reported whereby a tyrosine kinase inhibitor, CI‐1033, significantly enhanced ubiquitination in HER2 molecule together with the inhibition of kinase activity (Citri *et al*., 2002). These reports, along with our own experimental findings discussed in this manuscript, highlight the use of compounds that enhance drug‐induced ubiquitin modification to augment antineoplastic effects and warrant the use of these inhibitors in further studies. Nevertheless, the lack of efficacy in preclinical xenograft models suggests that additional work is needed for clinical development of belinostat in SCC tumors.

## Author contributions

LRK, SCL, WJC, and BCG conceived and designed the project. LRK, WRO, and CL acquired the data. LRK, TZT, HH, and PJE analyzed and interpreted the data. LRK, WJC, PJE, and BCG wrote and/or reviewed the manuscript. BCG supervised the study.

## Supporting information


**Fig. S1.** Belinostat and cisplatin inhibits colony formation in lung squamous cell carcinoma (SCC) cells.
**Fig. S2.** Belinostat treatment induces STAT3 signaling in Calu‐1 cells.
**Fig. S3.** Expressions of HDACs and sirtuin‐1 in normal lung and squamous cell carcinoma (SCC) cell lines.
**Fig. S4.** Belinostat increases Bim expression in lung SCC cells.
**Fig. S5.** Belinostat treatment induces acetylation of both Histone H3 and H4.
**Fig. S6.** Knockdown of FBXO3 and FBXW10 partially reduces belinostat sensitivity in H520 cells.
**Fig. S7.** Lack of *in vivo* efficacy of belinostat and cisplatin in lung squamous cell carcinoma (SCC) xenograft.

**Table S1.** Sequences for primers used in real‐time quantitative PCR.
**Table S2.** Computational and curated gene sets for ubiquitination‐related genes (GSEA c2, c4, and c5).
