# Supplementary material for: Belinostat exerts antitumor cytotoxicity through the ubiquitin‐proteasome pathway in lung squamous cell carcinoma
Source: Mol Oncol. 2017 May 30;11(8):965–80. doi: 10.1002/1878-0261.12064 (PMC5537703; doi:10.1002/1878-0261.12064)
Supplement: Supplementary file 1 — Fig. S1. Belinostat and cisplatin inhibits colony formation in lung squamous cell carcinoma (SCC) cells. Fig. S2. Belinostat treatment induces STAT3 signaling in Calu‐1 cells. Fig. S3. Expressions of HDACs and sirtuin‐1 in normal lung and squamous cell carcinoma (SCC) cell lines. Fig. S4. Belinostat increases Bim expression in lung SCC cells. Fig. S5. Belinostat treatment induces acetylation of both Histone H3 and H4. Fig. S6. Knockdown of FBXO3 and FBXW10 partially reduces belinostat sensitivity in H520 cells. Fig. S7. Lack of in vivo efficacy of belinostat and cisplatin in lung squamous cell carcinoma (SCC) xenograft. [file MOL2-11-965-s001.pdf]

Supplementary Figure S1

A

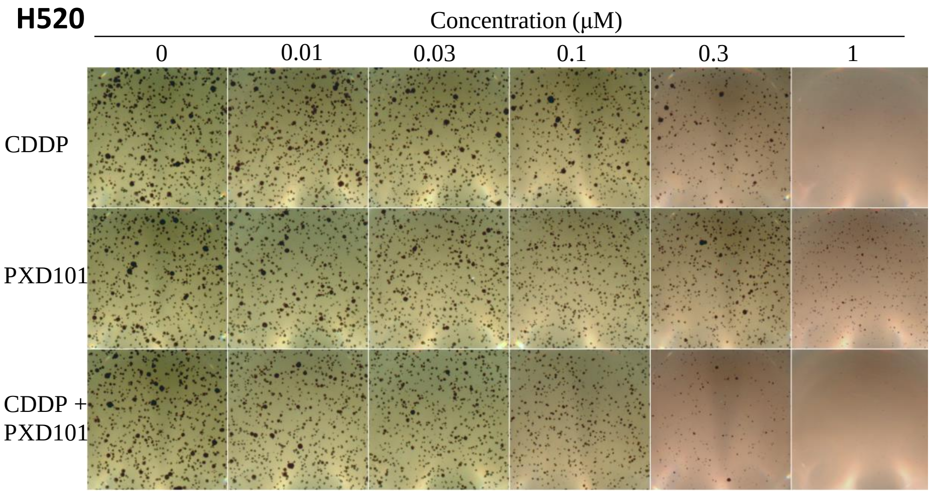

B

	CI (ED50-ED80)	
	**average**	**std. dev.**
**H2170**	0.82	0.18
**H520**	0.91	0.07
**ChaGo-k-1**	1.05	0.14
**Calu-1**	1.19	0.23

Supplementary Figure S1
Belinostat and cisplatin inhibits colony formation in lung squamous cell carcinoma (SCC) cells. Lung SCC lines were cultured in soft agar in the presence of increasing doses of belinostat (PXD101), cisplatin (CDDP) and in combination. **A**, representative images were shown to display dose-dependent colony inhibition H520 cells (*n* = 3). **B**, the combination index (CI) values of belinostat and cisplatin in soft agar assay between effective doses of 50-80% were calculated from isobologram analysis. Values were shown as average CI ± SD (*n* = 3). Combination index of CI < 0.8 indicates synergism, 0.8 < CI < 1.2 indicates additive effect, and CI > 1.2 indicates antagonism.

Supplementary Figure S2

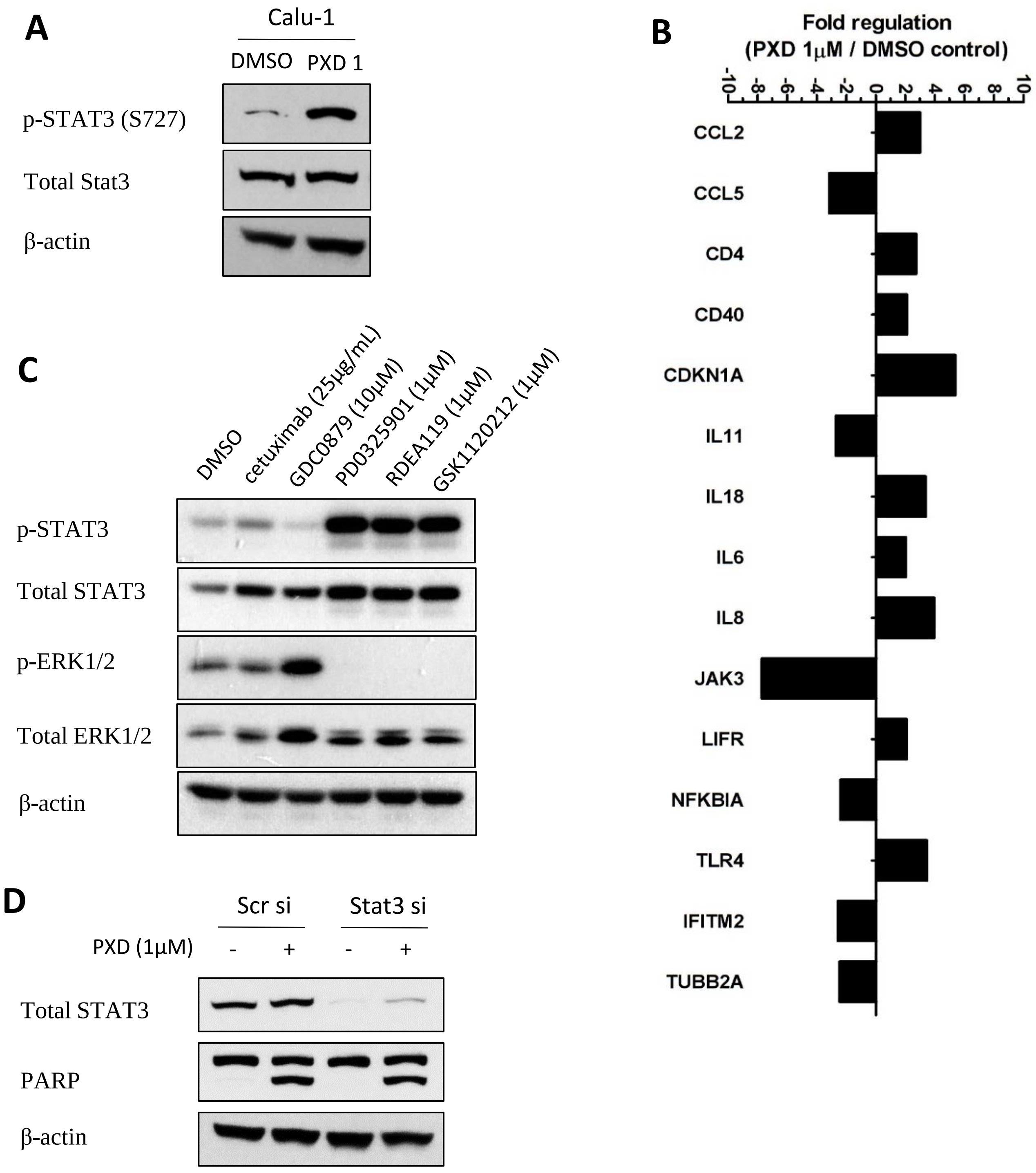

Supplementary Figure S2

Belinostat treatment induces STAT3 signaling in Calu-1 cells. **A**, cell lysates were harvested from Calu-1 cells after treatment with belinostat (1 μM) to evaluate the changes in STAT3 phosphorylation. **B**, Calu-1 cells were treated with vehicle and belinostat (1 μM) treatment. Total mRNA transcript levels of 84 key IL-6/STAT3 genes were assessed with RT^2 Profiler PCR Array. Data were presented as bar chart representing the relative value of the significantly altered genes (> 2 folds) as compared to vehicle (n = 1). **C**, the inhibitory effects of cetuximab, GDC0879, or MEK inhibitors (PD0325901, RDEA119, GSK1120212) on ERK and STAT3 phosphorylation were assessed by Western blotting in Calu-1 cells. **D**, immunoblots show the effects of STAT3 siRNA knockdown on affecting belinostat-induced apoptosis in Calu-1 cells. 50 nM siRNA was used per transfection. β-actin shown as loading control.

Supplementary Figure S3

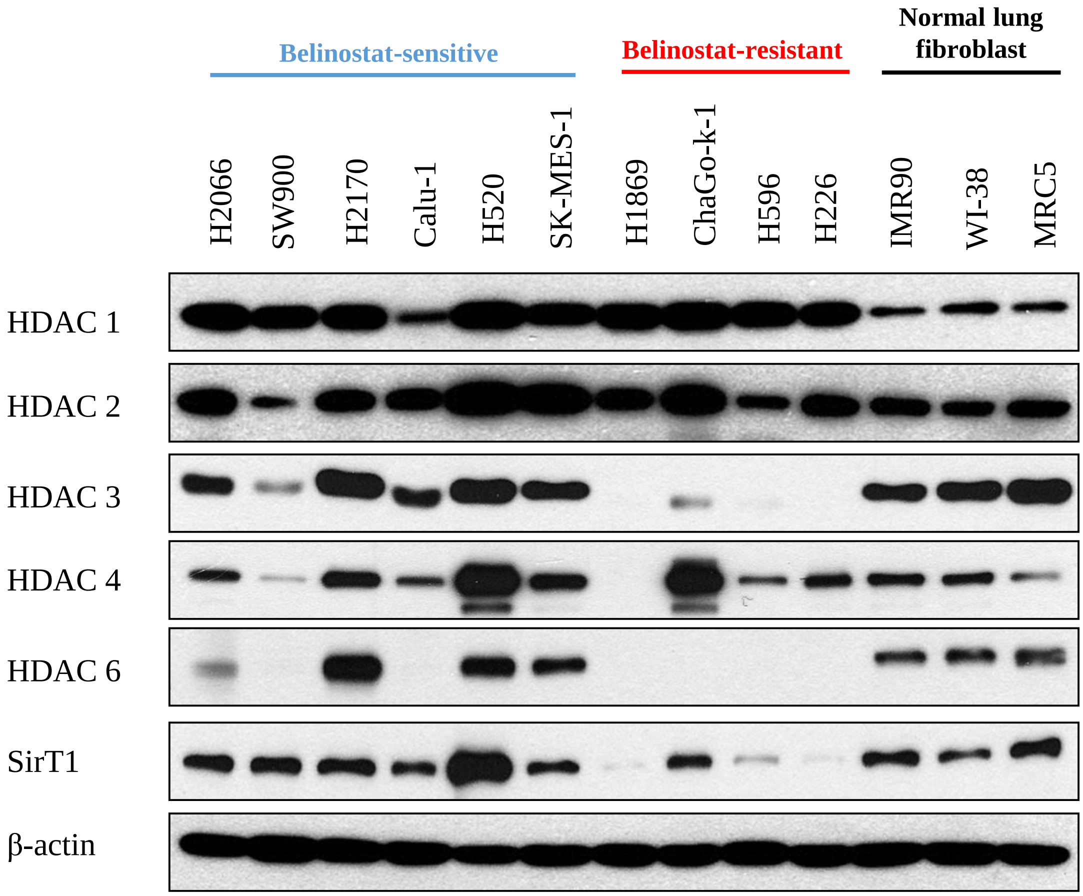

Supplementary Figure S3

Expressions of HDACs and sirtuin-1 in normal lung and squamous cell carcinoma (SCC) cell lines. Cell lysates were harvested from normal lung fibroblast cells, and both belinostat-sensitive and –resistant lung SCC cells. Immunoblots were performed to evaluate the expression of HDAC1, HDAC2, HDAC3, HDAC4, HDAC6 and sirtuin-1. β-actin shown as loading control.

Supplementary Figure S4

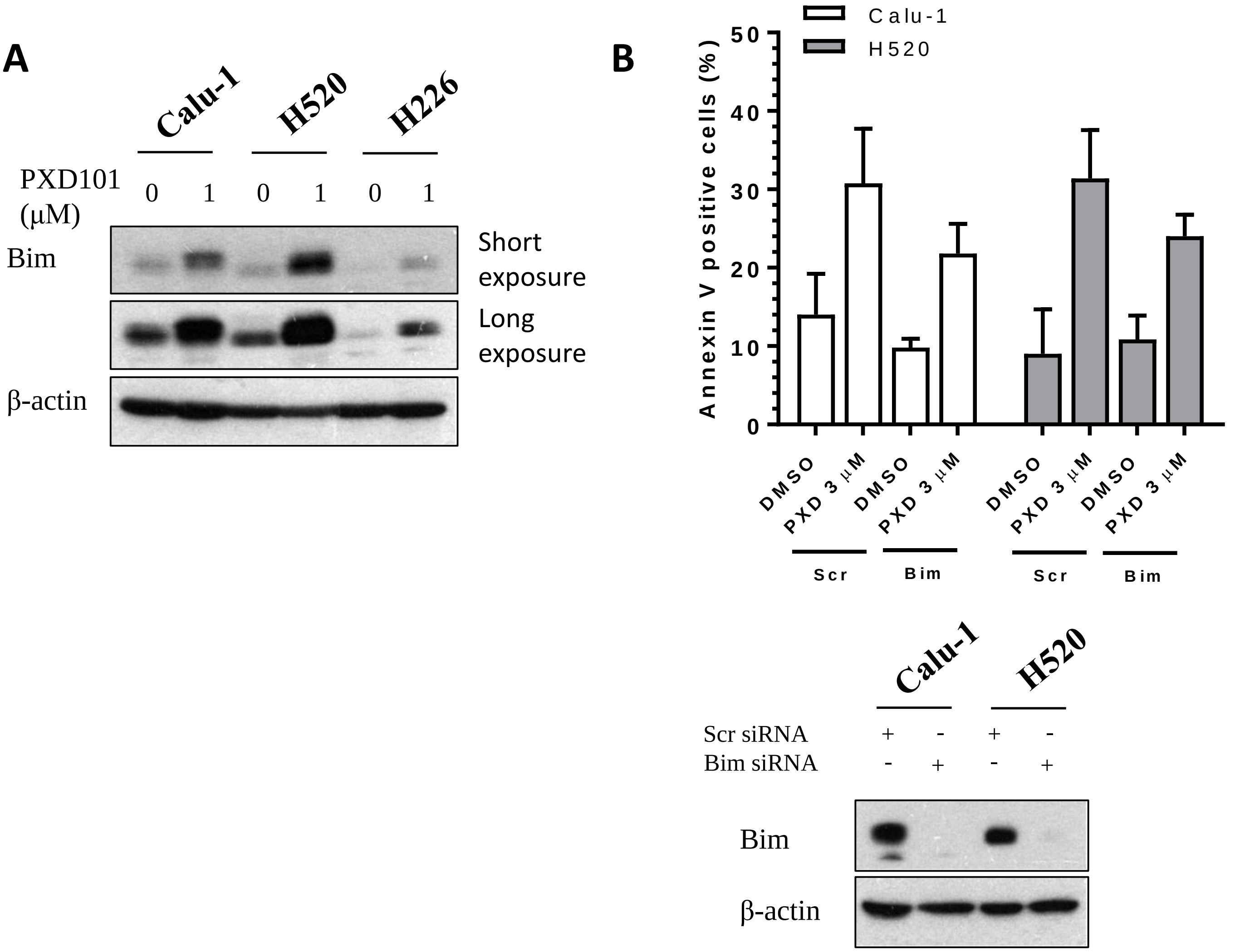

Supplementary Figure S4

Belinostat increases Bim expression in lung SCC cells. **A**, cell lysates were harvested from Calu-1, H520 and H226 cells after 48 hours treatment with belinostat (1 µM) to evaluate the changes in PARP and Bim. β-actin shown as loading control. **B**, Calu-1 and H520 were transfected with scrambled (Scr) or Bim siRNA, and treated with vehicle or belinostat (3 µM) for 48 hours before staining with Annexin-V/propidium iodide (PI). 50 nM siRNA was used per transfection. Immunoblots show the expression of Bim protein after siRNA transfection. The percentage of Annexin-V positive cells was shown as mean ± SD. *$P < 0.05$.

Supplementary Figure S5

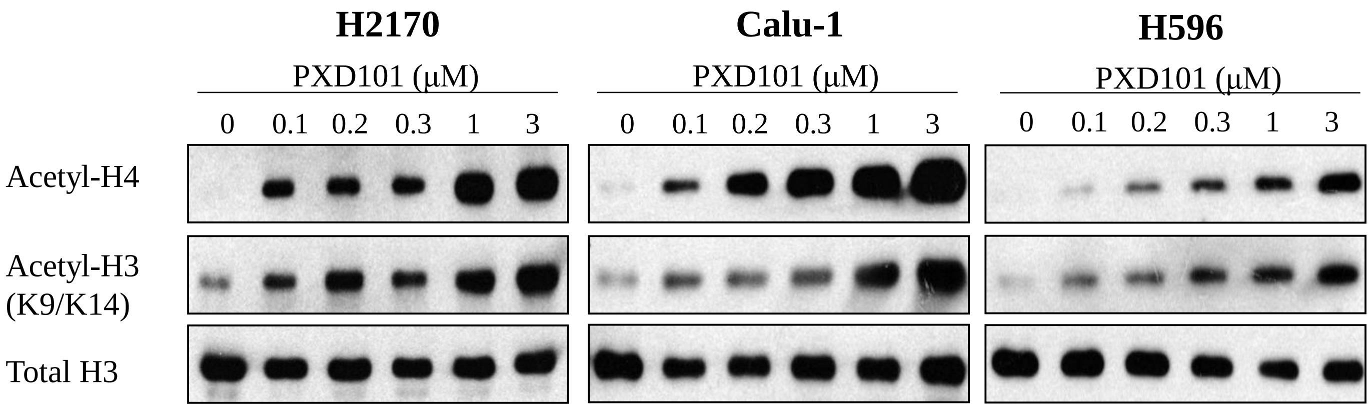

Supplementary Figure S5

Belinostat treatment induces acetylation of both Histone H3 and H4. SCC cells were treated with vehicle and increasing doses of belinostat (0.1, 0.2, 0.3, 1, 3μM). Histone proteins were isolated by acid extraction, and normalized according to protein concentration. Immunoblotting was performed to evaluate the changes in acetylated Histone H3 and H4 in H2170, Calu-1 and H596 cells. Total Histone H3 shown as loading control.

Supplementary Figure S6

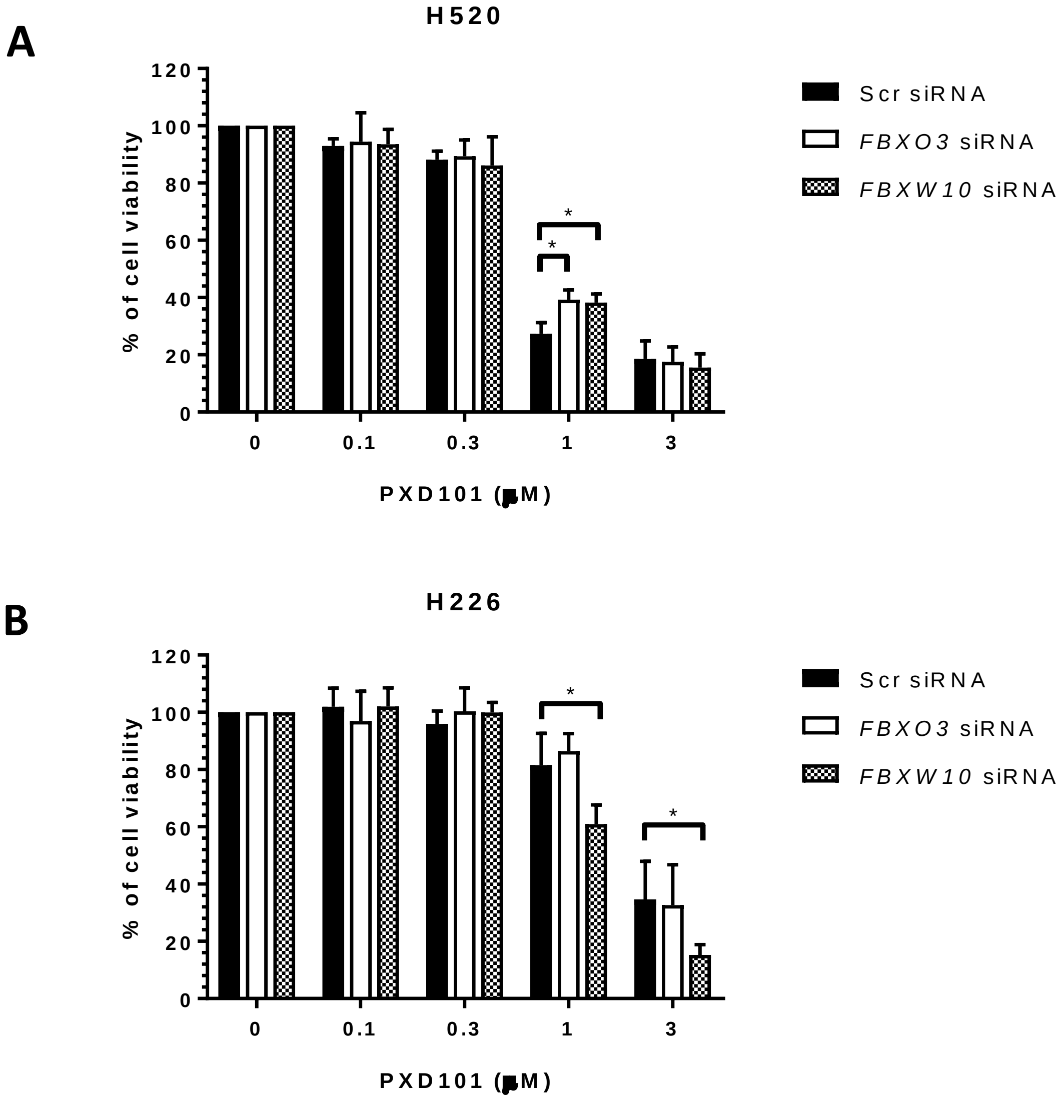

Supplementary Figure S6

Knockdown of FBXO3 and FBXW10 partially reduces belinostat sensitivity in H520 cells. H520 (A) and H226 (B) cell lines were transfected with scrambled (Scr), *FBXO3* or *FBXW10* siRNA, and treated with vehicle or increasing doses of belinostat (0.1, 0.3, 1, 3 μM) for 72 hours and cell viability was determined with CellTiter assay. Data are represented as mean percentage of cell viability ± SD (*n* = 3). 50 nM siRNA was used per transfection.

Supplementary Figure S7

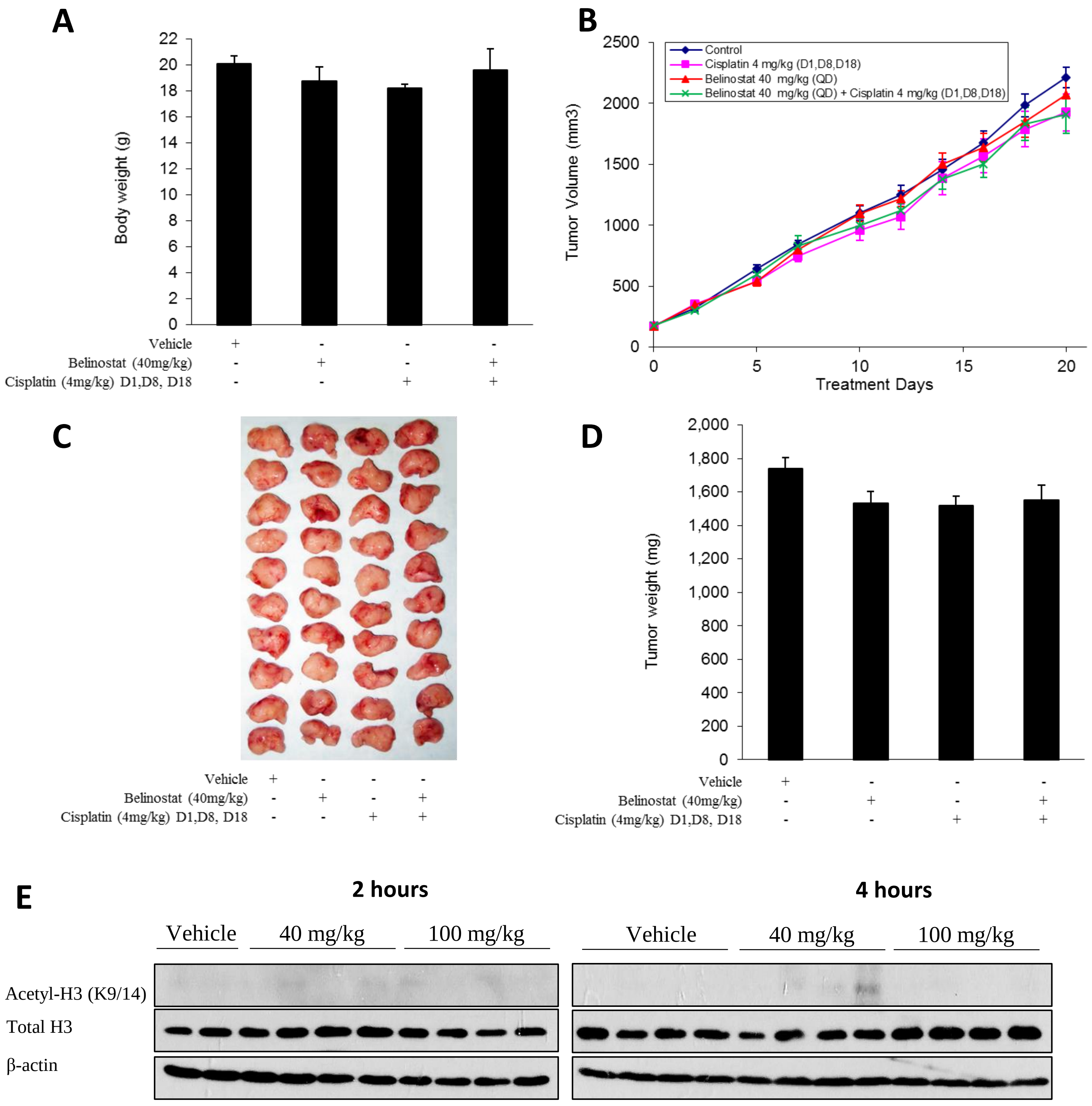

Supplementary Figure S7

Lack of *in vivo* efficacy of belinostat and cisplatin in lung squamous cell carcinoma (SCC) xenograft. Calu-1-derived xenografts were treated with vehicle, belinostat (40 mg/kg, 5 days weekly), cisplatin (5 mg/kg weekly), or a combination of belinostat and cisplatin for 21 days. **A**, change in body weight of mice at study endpoint was reported as mean ± SD (n = 10 tumors per group). **B**, change in tumor volume was monitored over 21 days and presented as mean ± SD (n = 10 tumors per group). **C**, images showing final tumor size for each individual tumor in respective treatment group were displayed, and the tumor weight (**D**) was presented as mean ± SD (n = 10 tumors per group). **E**, immunoblots showed levels of acetylated histone H3 and total H3 in independent tumor tissues. β-actin shown as loading control.
