# Supplementary material for: Belinostat exerts antitumor cytotoxicity through the ubiquitin‐proteasome pathway in lung squamous cell carcinoma
Source: Mol Oncol. 2017 May 30;11(8):965–80. doi: 10.1002/1878-0261.12064 (PMC5537703; doi:10.1002/1878-0261.12064)
Supplement: Supplementary file 2 — Table S1. Sequences for primers used in real‐time quantitative PCR. Table S2. Computational and curated gene sets for ubiquitination‐related genes (GSEA c2, c4, and c5). [file MOL2-11-965-s002.docx]

| **Gene name** | **Primer 1** | **Primer 2** |
| --- | --- | --- |
| *SOS1* | Pre-designed primers by TaqMan® Applied Biosystems. | |
| *SOS2* | Pre-designed primers by TaqMan® Applied Biosystems. | |
| *BRCA1* | 5'-ATACCTGCCTCAGAATTTCCTC-3' | 5'-AATGGAAGGAGAGTGCTTGG-3' |
| *FBXO3* | 5'-TCTTCAGAACGATAGTGATTAGACAG-3' | 5'-TCGATGTTCATACCGAATTCACA-3' |
| *FBXW10* | 5'-CTGGCTTGGTTATGTCTCCTT-3' | 5'-GCCCCAGAAAATGAACACTTG-3' |
| *UBE2C* | 5'-GCAGGTACTTCTTAAAAGCTGTG-3' | 5'-TCTCCATCCAGAGCCTTCT-3' |
| *PARK2* | 5'-ACTCGCAGCCACAGTTC-3' | 5'-GCCTCCAAAGAAACCATCAAG-3' |
| *RNF148* | 5'-CCCATTTGCTCCCTTCTCTG-3' | 5'-CATCCTTTGACCAATTTCAGCA-3' |
| *HECW1* | 5'-ACAGATTCTTCACACTACACAGG-3' | 5'-GCGTTTCTCATCAGCAGACT-3' |

**Supplementary Table S1**

Sequences for primers used in real time quantitative PCR.

| AMFR | CDC27 | FBXO21 | KLHL13 | NFX1 | RNF125 | SHPRH | TRIM28 | UBE2E2 | ZNRF1 |
| --- | --- | --- | --- | --- | --- | --- | --- | --- | --- |
| ANAPC10 | CDC34 | FBXO25 | KLHL2 | NFXL1 | RNF126 | SIAH1 | TRIM3 | UBE2E3 | ZNRF2 |
| ANAPC11 | CDC34 | FBXO3 | KLHL20 | NHLRC1 | RNF128 | SIAH2 | TRIM31 | UBE2G1 |  |
| ANAPC11 | CDH1 | FBXO3 | KLHL21 | NOSIP | RNF13 | SKP1 | TRIM32 | UBE2G2 |  |
| ANAPC13 | CGRRF1 | FBXO30 | KLHL22 | OSTM1 | RNF130 | SKP1 | TRIM33 | UBE2H |  |
| ANAPC2 | CHFR | FBXO31 | KLHL24 | PARK2 | RNF135 | SKP2 | TRIM36 | UBE2I |  |
| ANAPC2 | CISH | FBXO31 | KLHL41 | PARK2 | RNF138 | SKP2 | TRIM37 | UBE2J1 |  |
| ANAPC4 | CNOT4 | FBXO32 | KLHL7 | PAX6 | RNF139 | SMURF1 | TRIM38 | UBE2J2 |  |
| ANAPC5 | CRBN | FBXO33 | KLHL9 | PCGF2 | RNF14 | SMURF1 | TRIM39 | UBE2K |  |
| ANAPC7 | CUL1 | FBXO4 | LMO7 | PCGF3 | RNF144A | SMURF2 | TRIM4 | UBE2L3 |  |
| ANKIB1 | CUL1 | FBXO4 | LNX1 | PCGF5 | RNF144B | SMURF2 | TRIM40 | UBE2M |  |
| APC2 | CUL2 | FBXO43 | LONRF1 | PDZRN3 | RNF145 | SOCS1 | TRIM41 | UBE2N |  |
| ARIH1 | CUL2 | FBXO44 | LRRC41 | PDZRN4 | RNF146 | SOCS2 | TRIM44 | UBE2Q1 |  |
| ARIH1 | CUL3 | FBXO5 | LRSAM1 | PELI1 | RNF148 | SOCS3 | TRIM45 | UBE2R2 |  |
| ARIH2 | CUL3 | FBXO6 | LTN1 | PELI2 | RNF150 | SOCS4 | TRIM48 | UBE2S |  |
| ASB1 | CUL4A | FBXO7 | MALT1 | PEX12 | RNF152 | SOCS5 | TRIM5 | UBE2T |  |
| ASB18 | CUL4A | FBXO9 | MAP3K1 | PEX2 | RNF166 | SOCS6 | TRIM52 | UBE2W |  |
| ASB2 | CUL4B | FBXW10 | MARCH1 | PHIP | RNF167 | SPOP | TRIM54 | UBE2Z |  |
| ASB3 | CUL4B | FBXW10 | MARCH11 | PHRF1 | RNF168 | SPSB1 | TRIM56 | UBE3A |  |
| ASB9 | CUL5 | FBXW11 | MARCH2 | PIAS1 | RNF170 | SPSB2 | TRIM61 | UBE3B |  |
| ATG7 | CUL5 | FBXW12 | MARCH3 | PIAS2 | RNF175 | SPSB4 | TRIM62 | UBE3C |  |
| ATRX | CUL7 | FBXW2 | MARCH5 | PIAS4 | RNF181 | STUB1 | TRIM63 | UBE4A |  |
| BARD1 | CUL7 | FBXW4 | MARCH6 | PJA1 | RNF187 | STUB1 | TRIM64 | UBE4B |  |
| BARD1 | CUL9 | FBXW5 | MARCH7 | PJA2 | RNF19A | SYVN1 | TRIM67 | UBE4B |  |
| BCOR | DCST1 | FBXW7 | MARCH8 | PML | RNF19B | SYVN1 | TRIM68 | UBR1 |  |
| BFAR | DDA1 | FBXW9 | MDM2 | RAD18 | RNF2 | TCEB1 | TRIM71 | UBR1 |  |
| BIRC2 | DDB1 | FEM1B | MDM2 | RAD51 | RNF20 | TCEB2 | TRIM72 | UBR2 |  |
| BIRC3 | DDB1 | GAN | MDM4 | RANBP2 | RNF207 | TMEM189 | TRIM73 | UBR2 |  |
| BIRC7 | DDB2 | HACE1 | MEX3A | RBBP6 | RNF208 | TNFAIP1 | TRIM74 | UBR3 |  |
| BMI1 | DET1 | HECTD1 | MEX3C | RBCK1 | RNF212 | TNFAIP3 | TRIM75 | UBR4 |  |
| BRAP | DIABLO | HECTD3 | MEX3D | RBX1 | RNF215 | TOPORS | TRIM8 | UBR5 |  |
| BRCA1 | DTL | HECW1 | MGRN1 | RC3H1 | RNF216 | TP53 | TRIM9 | UBR7 |  |
| BRCA1 | DTX3 | HECW1 | MIB1 | RC3H2 | RNF219 | TRAF2 | TRIML1 | UHRF2 |  |
| BRCA2 | DTX4 | HECW2 | MIB1 | RCHY1 | RNF220 | TRAF3 | TRIP12 | UNK |  |
| BRCC3 | DZIP3 | HERC1 | MIB2 | RFPL3 | RNF24 | TRAF4 | TSPAN17 | VHL |  |
| BRCC3 | DZIP3 | HERC2 | MID1 | RFWD2 | RNF26 | TRAF5 | TTC3 | VHL |  |
| BTBD1 | ENC1 | HERC3 | MID2 | RFWD2 | RNF31 | TRAF6 | UBA1 | VprBP |  |
| BTBD2 | FANCL | HERC4 | MKRN1 | RHOBTB1 | RNF34 | TRAF7 | UBA2 | VPS11 |  |
| BTRC | FBXL12 | HERC5 | MKRN2 | RHOBTB3 | RNF38 | TRAIP | UBA3 | VPS18 |  |
| BTRC | FBXL14 | HERC5 | MNAT1 | RING1 | RNF4 | TRIM10 | UBA5 | VPS41 |  |
| CBL | FBXL15 | HERC6 | MOCS3 | RNF10 | RNF40 | TRIM11 | UBA6 | VPS8 |  |
| CBL | FBXL18 | HLTF | MSL2 | RNF103 | RNF41 | TRIM13 | UBB | WDSUB1 |  |
| CBLB | FBXL2 | HUWE1 | MUL1 | RNF11 | RNF43 | TRIM17 | UBC | WHSC1 |  |
| CBLC | FBXL20 | HUWE1 | MYCBP2 | RNF111 | RNF44 | TRIM2 | UBE2A | WSB1 |  |
| CBLL1 | FBXL3 | IRAK1 | MYLIP | RNF113A | RNF5 | TRIM22 | UBE2B | WWP1 |  |
| CCNB1IP1 | FBXL4 | IRF2BPL | NAE1 | RNF114 | RNF6 | TRIM23 | UBE2C | WWP1 |  |
| CCNF | FBXL5 | ITCH | NEDD4 | RNF115 | RNF7 | TRIM24 | UBE2D1 | XIAP |  |
| CDC16 | FBXO11 | KBTBD7 | NEDD4L | RNF121 | RNF8 | TRIM25 | UBE2D2 | ZBTB16 |  |
| CDC20 | FBXO18 | KCTD10 | NEDD8 | RNF123 | RSPRY1 | TRIM26 | UBE2D3 | ZER1 |  |
| CDC23 | FBXO2 | KEAP1 | NEURL1 | RNF123 | SAE1 | TRIM27 | UBE2E1 | ZNF645 |  |

**Supplementary Table S2:**

Computational and curated gene sets for ubiquitination-related genes (GSEA c2, c4, and c5).
